# Pre-operative Hemostatic Status in Dogs Undergoing Splenectomy for Splenic Masses

**DOI:** 10.3389/fvets.2022.686225

**Published:** 2022-04-25

**Authors:** Jourdan B. McPhetridge, Alex M. Lynch, Cynthia R. L. Webster, Emily McCobb, A. M. de Laforcade, Therese E. O'Toole

**Affiliations:** ^1^Department of Clinical Sciences, North Carolina State University, Raleigh, NC, United States; ^2^Department of Clinical Sciences, Cummings School of Veterinary Medicine, Tufts University, Grafton, MA, United States

**Keywords:** TEG, splenectomy, splenic mass, dog, hypercoagulability

## Abstract

Portal system thrombosis is a rare but potentially fatal complication of splenectomy in dogs. The mechanism behind development of post-operative portal system thrombosis is unclear but may include alterations of portal blood flow following surgery, acquired hypercoagulability and endothelial dysfunction. The aim of the study was to evaluate hemostatic biomarkers in hemodynamically stable (heart rate <130 beats/min, blood lactate < 2.5 mMol/L) and non-anemic (hematocrit >35%) dogs prior to splenectomy for splenic masses. Our hypothesis was that this population of stable dogs would have pre-existing laboratory evidence of hypercoagulability unrelated to shock, bleeding, anemia, or other pre-operative comorbidities. Pre-operatively, abdominal ultrasonography was performed and blood was collected for platelet enumeration, prothrombin time (PT), activated partial thromboplastin time (aPTT), kaolin-activated thromboelastography (TEG), fibrinogen, von Willebrand factor activity (vWF:Ag), antithrombin and thrombin-antithrombin complex (TAT). Histopathological diagnosis and 30-day survival were recorded. None of the 15 enrolled dogs had pre-operative sonographic evidence of portal system thrombosis. Three of fifteen dogs were thrombocytopenic, three had thrombocytosis, three were hyperfibrinogenemic, one had low vWF:Ag, three had mild prolongations of PT and none had abnormal aPTT. Based on the TEG G value, 13/15 dogs were hypercoagulable (mean ± SD 13.5 ± 5.4 kd/s). Antithrombin deficiency was identified in 9/15 dogs (mean ± SD 68.7 ± 22.7%) with 5/9 having concurrently elevated TAT suggesting active thrombin generation. No dogs developed portal system thrombosis and all achieved 30-day survival. Pre-operative hypercoagulability was recognized commonly but its association with post-operative thrombosis remains undetermined.

## Introduction

Splenectomy is commonly performed in dogs with splenic masses either in the presence or absence of spontaneous hemoperitoneum. The peri-operative mortality rate for dogs undergoing splenectomy is reported between 8 and 33% (1–5). In one study of 539 dogs undergoing splenectomy for splenic masses, 41 dogs (8%) died or were euthanized during or following surgery (5). Mortality in 10/41 (24%) of these dogs was associated with hemorrhage and hemostatic dysfunction, while post-operative thrombosis [portal system thrombosis (PST) or pulmonary thromboembolism] was suspected in 13/41 (32%) dogs that experienced unexpected peri-operative deaths. Thrombosis was definitely confirmed in 2 of these 13 cases. Portal system thrombosis is reported to occur between 5 and 10% of people following splenectomy (6–8). The sequelae to acute PST can lead to significant morbidity and mortality (9). Pre-operative prediction of post-splenectomy thrombotic complications in dogs would be desirable but is challenging and will require a better understanding of the mechanisms associated with the development of post-splenectomy PST. Factors that have been implicated in people include altered portal blood flow following ligation of splenic vessels and intrinsic hemostatic derangements (7, 10).

Variable hemostatic profiles have been previously reported in dogs with splenic masses. Some dogs with hemoperitoneum secondary to splenic masses, for example, have pre-operative evidence of hypocoagulability and hyperfibrinolysis on thromboelastography (TEG) (11). Hypercoagulability has also been identified in dogs with a variety of neoplastic processes including hemangiosarcoma (12–14). In one study of 34 dogs undergoing splenectomy for splenic masses, post-operative thrombocytosis was commonly identified along with TEG variables suggestive of hypercoagulability (shortened K time and increased angle, maximum amplitude (MA) and G) (15). Increased platelet number may be a risk factor for post-splenectomy thrombosis in people (10). It is unknown whether subclinical pre-operative hemostatic derangements may be present in some dogs with splenic masses that in turn might contribute to the development of unexpected thrombotic events following splenectomy. To the authors' knowledge, comprehensive characterization of the pre-operative hemostatic status of dogs with splenic masses, without evidence of hemodynamic instability or comorbidities associated with hypercoagulability, has not been investigated. Understanding this population of dogs with splenic masses, without confounding factors that may impact their hemostatic status, would be useful to determine predictors of post-splenectomy thrombosis. Pre-existing hypercoagulability may set the stage for additional events to merge in the pathogenesis of thrombosis. Clinically, it would also be advantageous to identify dogs that may benefit from the administration of antithrombotic drugs.

The aim of this prospective study therefore was to evaluate pre-operative hemostatic status in a population of dogs with splenic masses. To limit the influence of certain conditions known to alter hemostasis, a study population restricted to dogs without hemodynamic instability or comorbidities associated with hypercoagulability would be selected. Our hypothesis was that this population of stable dogs would have pre-existing laboratory evidence of hypercoagulability unrelated to shock, bleeding, anemia, or other comorbidities commonly described as prothrombotic prior to surgery. An additional aim was to compare the hemostatic profiles of dogs that developed post-operative thrombosis to those that did not.

## Materials and Methods

### Animals

Client-owned dogs undergoing splenectomy for splenic masses at the Foster Hospital for Animals at the Cummings School of Veterinary Medicine at Tufts University were eligible for enrollment over a 12-month period (March 2014–March 2015). This study was approved by the Cummings School of Veterinary Medicine at Tufts University Clinical Science Research Committee institutional review board and client consent was obtained before patient enrollment. Dogs were eligible for inclusion if they had evidence of hemodynamic stability at presentation without the need for fluid resuscitation, defined as heart rate <130 beats per min and blood lactate <2.5 mmol/L, and were not anemic [packed cell volume (PCV) > 35%]. Animals were excluded if they had evidence of concurrent disease known to be associated with hypercoagulability including protein-losing nephropathy (16), protein-losing enteropathy (17), hypercortisolism (18), hepatopathy (19, 20), acute pancreatitis (21); immune-mediated diseases (22) and cardiac disease (23). Likewise, dogs that had received oral, parenteral or topical corticosteroids or medications that could predispose to hypocoagulability (e.g., non-steroidal anti-inflammatory drugs, platelet inhibitors, omega 3 fatty acids) within the last 2-months were excluded. The decision to exclude patients was made based on expert opinion (e.g., board-certified veterinary criticalist and/or internist) after review of medical records, which included the results of pre-operative hematology and serum chemistry profiles and urinalysis. Pre-operative abdominal ultrasound was performed in each dog by an experienced operator (board-certified veterinary radiologist or a radiology resident under their supervision). Splenectomy was performed by the soft tissue surgery service at the Foster Hospital for Animals at the Cummings School of Veterinary Medicine at Tufts University by a board-certified surgeon or resident under their supervision. Patients were monitored for clinical signs that could be attributed to post-operative thrombosis during hospitalization and owners were instructed to continue monitoring for these signs following discharge for 30 days. These signs included an acute onset of abdominal pain, tachypnea, cardiovascular collapse secondary to gastrointestinal losses, vomiting, and development of abdominal distension due to effusion (9). Histopathologic examination of the splenic masses was performed by a board-certified anatomic pathologist at the Cummings School of Veterinary Medicine at Tufts University.

### Blood Collection and Handling for Analysis

A total of 6 mL of blood was collected *via* direct atraumatic venipuncture of a peripheral vein using an 18-gauge vacutainer blood collection needle. This blood was collected sequentially into plastic tubes containing 3.2% sodium citrate (9:1 whole blood: citrate), lithium heparin (17 IU/mL), and 7.5 % potassium EDTA, then agitated gently. Heparinized blood samples were immediately used for blood lactate quantification performed on a point-of-care lactate monitor^1^ and for determination of a spun PCV and total solids (TS) by refractometer. A platelet count was performed on the EDTA whole blood using an automated hematology analyzer^2^ and verified by direct blood smear evaluation performed by a hematology laboratory technician.

Following a 30-min rest period at room temperature (~23°C), a kaolin-activated TEG^3^ was performed on an aliquot of the citrated whole blood as previously described (24, 25). Briefly, 1 mL of citrated whole blood was added to a vial of kaolin^4^,^5^ and mixed gently. Then, 340 μL of this mixed sample was added to a standard TEG pin and cup containing 20 μL of calcium chloride that had been pre-warmed to 37°C. Each TEG was run by the same experienced laboratory technician. The TEG variables [R time, K time, alpha angle, maximum amplitude (MA), G and LY60%] were recorded after a 90-min run time. Hypercoagulability was defined as a TEG G value higher than the institutional reference interval (G > 8.5 k dynes/s) consistent with previous publications (9, 19, 20).

The remaining citrated whole blood was centrifuged at 4,000 × g for 15 min at room temperature to yield platelet poor plasma (PPP) and packed red cells. The PPP was harvested and frozen at −80°C for batch analysis of hemostatic biomarkers within 6 months. Fibrinogen, prothrombin time (PT), activated partial thromboplastin time (aPTT), von Willebrand factor antigen assay (Vwf:Ag), and antithrombin activity (AT) were used performing an automated analyzer. These assays were calibrated using a 3-point dilution standard curve using pooled canine plasma collected from 32 healthy dogs at the Cummings School of Veterinary Medicine at Tufts University. Thrombin-antithrombin complex (TAT) concentrations were measured in duplicate using a combination sandwich enzyme immunoassay previously validated in dogs.^6^

A reference population of 75 normal dogs were used as controls. These healthy dogs were historical controls, and not specifically sex, age, or breed matched to the dogs with splenic masses. The reference intervals for the hemostatic tests performed were determined by the Coagulation Laboratory at the Foster Hospital for Animals at the Cummings School of Veterinary Medicine at Tufts University in accordance with established guidelines from the American College of Veterinary Clinical Pathology (26). The normal dogs ranged in age from 1 to 14.1 years, with a mean ± SD of 4.5 ± 2.8 years. Breeds included mixed breed (*n* = 20), Labrador retriever (*n* = 14), German shepherd (*n* = 8), Staffordshire terrier (*n* = 5), German short haired pointer (*n* = 4), Golden retriever (*n* = 4), American bulldog (*n* = 2), Irish setter (*n* = 2), Dachshund (*n* = 2), Lhasa apso (*n* = 2) and one each of 12 additional breeds. The sex distribution of this population was 23% female, 26% spayed female, 15% male and 36% castrated male. All dogs were owned by the students, staff, or faculty of the Cummings School of Veterinary Medicine at Tufts University. These dogs were deemed to be healthy based on no history of illness within the previous 6 months, normal physical examination, complete blood count, chemistry profile, urinalysis, PT, and aPTT prior to blood collection. Diagnostic imaging was not used for routine screening, however. Control dogs were not receiving any medications, other than routine heartworm preventative and flea and tick control and were all up to date with routine vaccinations. Sample collection, processing and analysis of samples strictly followed the aforementioned technique used for the dogs with splenic masses. Histograms and box and whisker plots were performed, and the data was determined to be normally distributed. The reference interval was defined as the mean ± 2 standard deviations.

### Statistical Analysis

Shapiro-Wilk tests were used to evaluate normality of datasets. Parametric data are presented as mean ± standard deviation and were compared using the Student's *t*-test. Non-parametric data are presented as median (range) and were compared using Mann-Whitney Rank Sum tests. Significance was set at *P* < 0.05. All statistics were performed by a statistician using statistical software packages.^7^,^8^

## Results

In total, 18 dogs met the inclusion criteria, but 3 were excluded to concurrent conditions associated with hypercoagulability, leaving 15 dogs for inclusion in the final analysis. Median patient age was 11 years (range 5–14 years) and sex distribution included 11 castrated males, 3 spayed females, and 1 intact female. The mean weight was 31.5 ± 14.7 kg. Breeds represented included Labrador retriever (3/15), German shepherd dog (2/15), Rhodesian ridgeback (2/15), Border collie (2/15), Australian cattle dog (1/15), boxer (1/15), Jack Russell terrier (1/15), Newfoundland (1/15), Rottweiler (1/15), and Yorkshire terrier (1/15). All 15 dogs had presence of a splenic mass or masses confirmed by abdominal ultrasound. Thirteen of 15 (87%) dogs had abdominal masses identified incidentally during routine annual examinations. The remaining two dogs had splenic masses identified by abdominal ultrasound during their diagnostic work up for abdominal distension and mixed serum liver enzyme elevations, respectively. A solitary splenic mass was identified with ultrasound in 9 of 15 dogs (60%), and multiple splenic masses in the remaining 6/15 dogs (40%). Scant abdominal effusion was identified in 4 of the 15 dogs (27%). The portal vein was clearly visualized in 9 of the 15 dogs (60%). Visualization of the portal vein was obscured by the splenic mass itself in 5/15 (33%) dogs and by gastrointestinal gas shadowing in 1/15 (7%) dog. None of the dogs had pre-operative evidence of clinically significant bleeding or thrombosis, nor evidence of abdominal or pulmonary metastasis on pre-operative imaging.

**Figure 1:**
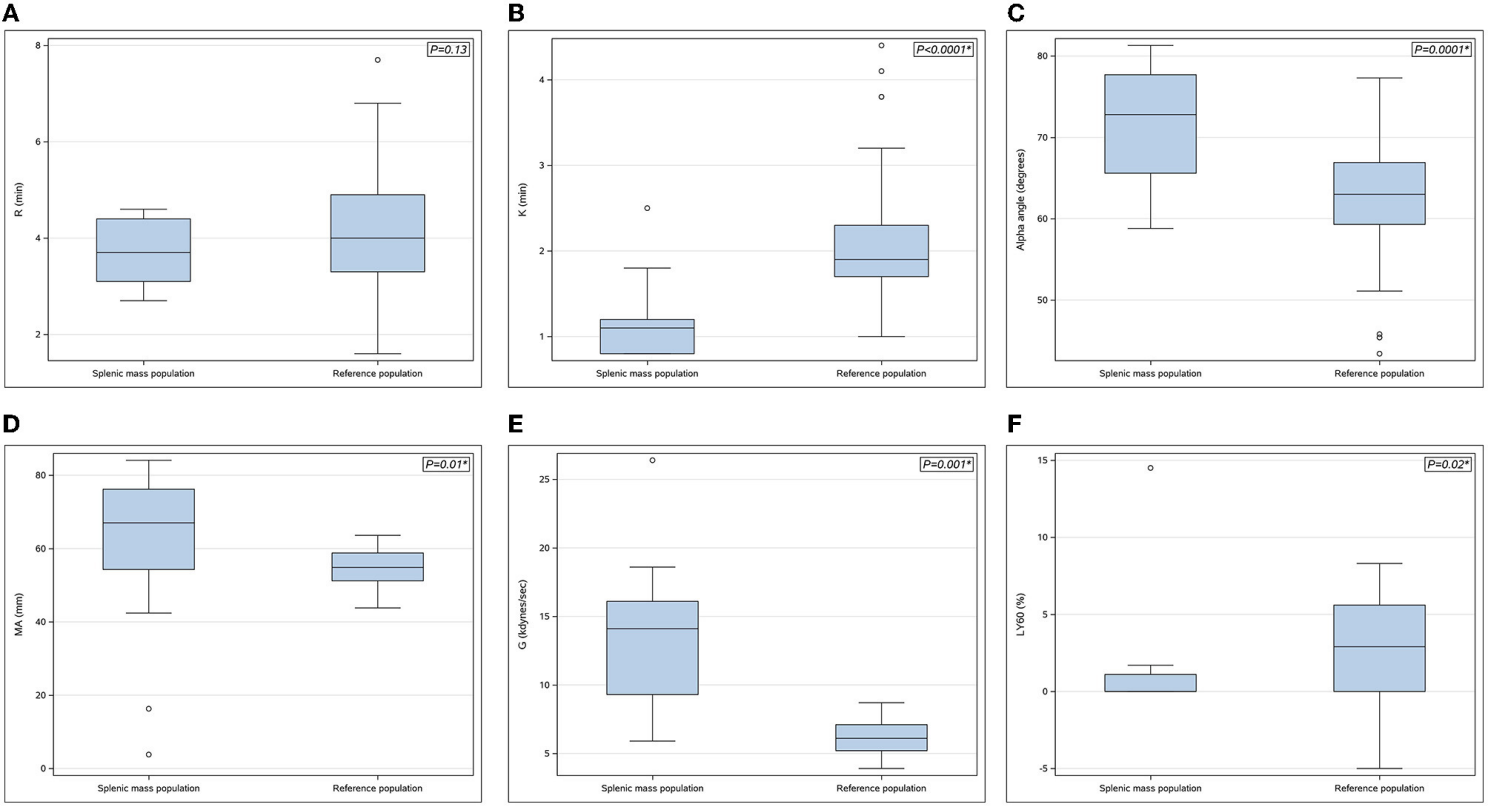
**(A–F)** Boxplots of thromboelastography parameters in dogs with splenic masses (*n* = 15) compared with the reference population (*n* = 75). **(A)** R-time (min), **(B)** K-time (min), **(C)** Alpha angle (degrees), **(D)** Maximum amplitude (MA) (mm), **(E)** G-value (kdynes/sec), **(F)** % lysis at 60 min (LY60%) (%). Each box represents the interquartile range (25th to 75th percentile) with the horizontal line within each box corresponding to the median value for each parameter. Whiskers represent the upper and lower limits of the range. Open circles represent outlier values. *P*-values are shown in the upper right-hand corner with statistical significance denoted by*.

The median baseline PCV and TS were 0.42 L/L (range 0.37–0.5 L/L) and 64 g/L (58–80 g/L), respectively. The mean pre-operative blood lactate concentration was 1.5 ± 0.5 mmol/L. The results of the hemostatic tests performed are presented in Table 1. Thrombocytopenia and thrombocytosis were identified in 3 of 15 dogs (20%) and 3 of 15 dogs (20%) with splenic masses, respectively. Three of 15 dogs (20%) had mild prolongations of PT but no abnormalities in aPTT were identified. Hyperfibrinogenemia was identified in 3/15 (20%) splenic mass dogs. One dog with a splenic mass had a vWF:Ag below reference range. There was no significant difference in platelet count, PT, aPTT, fibrinogen and vWF:Ag between dogs with splenic masses and the reference population. Antithrombin activity was significantly decreased in dogs with splenic masses compared to the healthy population. Antithrombin deficiency was present in 9/15 (60%) dogs with splenic masses, 5/9 (56%) of which had concurrently elevated plasma TAT complex concentrations. Thromboelastography parameters in dogs with splenic masses and normal dogs are presented in Figure 1. The K-time was significantly shorter and the alpha angle significantly increased in the splenic mass cohort. Indicators of clot strength (MA and G) were also significantly increased in dogs with splenic masses compared to the healthy dogs. Based on the *G-*value, 13/15 dogs (87%) with splenic masses were considered hypercoagulable. Dogs with splenic masses appeared significantly hypofibrinolytic compared to the reference population based on a significantly lower LY60%.

**Table 1:** Pre-operative hemostatic parameters in dogs with splenic masses compared to the healthy reference population.

**Variable**	**Splenic mass dogs Median (range)**	**Healthy reference population** **Median (range)**	***P*-value**
Platelet count (×10^9^/L)	295 (104–637)	353 (180–525)	0.83
PT (s)	7.7 (6.3–12.9)	7.0 (5–9)	0.06
aPTT (s)	12.5 (11.4–14.6)	15.2 (9.9–20.4)	0.11
Fibrinogen (g/L)	3.4 (1.7–7.2)	2.4 (7.3–4.1)	0.07
Antithrombin (%)	62.8 (36.2–114)	93 (75–110)	0.006
vWF:Ag (%)	90.2 (36.5–129)	90 (43–141)	0.74
TAT (μg/L)	2.7 (0.2–16.1)	2.1 (0.1–3.8)	0.09

*aPTT, activated partial thromboplastin time; PT, prothrombin time; TAT, thrombin antithrombin; vWF:Ag, von Willebrand factor antigen*.

Histopathology identified malignant neoplasia in 8/15 cases (53%) and benign lesions in 7/15 cases (47%). The 8 malignant tumors were undifferentiated sarcomas [4/8 (50%)], hemangiosarcoma [3/8 (38%)], and lymphoma [1/8 (13%)]. The 7 benign lesions consisted of haematoma [4/7 (57%)], lymphoid hyperplasia [2/7 (29%)], and nodular hyperplasia [1/7 (14%)]. None of the dogs with splenic mass lesions developed detectable acute thrombotic complications, therefore differences between dogs with respect to thrombosis development could not be evaluated. Every dog undergoing splenectomy achieved 30-day survival.

## Discussion

Pre-operative viscoelastic changes consistent with hypercoagulability were commonly recognized in this population of stable dogs with splenic masses. Dogs with splenic masses had TEG changes consistent with faster clot formation, increased clot strength and decreased fibrinolysis compared to normal dogs. This study supports the hypothesis that the presence of a splenic mass may contribute to pre-existing hypercoagulability that is unrelated to shock, bleeding, surgery, anemia, or other comorbidities commonly described as prothrombotic. There were no significant changes in hemostatic parameters between dogs with benign and malignant masses. Despite the TEG changes consistent with relative hypercoagulability and hypofibrinolysis, none of the dogs investigated here ultimately developed detectable or clinically evident post-splenectomy thrombosis.

Post-operative thrombosis is an uncommon yet potentially devastating complication of splenectomy in dogs with splenic masses (5, 9). However, in a study of peri-operative mortality in dogs undergoing splenectomy for splenic masses, acute portal system thrombosis and pulmonary thromboembolism were suspected in 9/41 (22%) and 4/41 (9.8%) deaths, respectively (5). Hypocoagulability and hyperfibrinolysis have previously been reported in dogs with splenic masses (11) but less is known regarding the prothrombotic tendency of some dogs with splenic masses prior to splenectomy. This may, in part, be related to the difficulties in assessing hypercoagulability and detecting microthrombi in clinical veterinary patients. Traditional coagulation tests (e.g., PT, aPTT) have typically been used for identification of hypocoagulability associated with clotting factor derangements. One retrospective study did find that dogs with shortened PT and aPTT were significantly more likely to have thrombosis, however (27). The increased availability of viscoelastic testing in veterinary medicine in recent years has provided a tool to gain further insight into the role of hypercoagulability in disease states in dogs (16–22).

In a recent study of dogs undergoing splenectomy, post-operative thrombocytosis and TEG variables suggestive of hypercoagulability (shortened K time, and increased angle, MA, and G) were reported (15). Interestingly, pre-operative thrombocytosis was only documented in 3% of dogs despite 45% having ≥1 TEG variable consistent with hypercoagulability, suggesting that a pre-operative hypercoagulable tendency in dogs with splenic masses is likely driven by factors other than platelet enumeration. Exclusion criteria in that study were limited to treatment with corticosteroids and non-steroidal anti-inflammatories and/or a diagnosis of hyperadrenocorticism. The data presented in the current study demonstrate that pre-existing hypercoagulability is common in dogs undergoing splenectomy for splenic masses, and our attempt to more stringently control for factors associated with hypercoagulability corroborates and further expands on the findings of Phipps et al. Despite demonstration of abnormal perioperative TEG variables consistent with hypercoagulability, no dogs in either study developed clinically detectable post-operative thrombosis, suggesting additional factors likely contribute to its development *in-vivo*. Subclinical thrombosis events could have occurred in some dogs described in the present study but gone undetected since post-operative diagnostic imaging was not performed. Since thrombotic complications following splenectomy are rare, it is plausible that a much larger population of dogs would be necessary to capture post-operative thrombotic events. It is also possible that post-operative thrombosis risk is enhanced in dogs with splenic masses not captured within our inclusion criteria—e.g., dogs with cardiovascular collapse secondary to hemorrhage; dogs with concurrent diseases associated with hypercoagulability.

The pathogenesis of PST in people following splenectomy is more fully, yet still incompletely, characterized compared to dogs (6–8, 10). One potential risk factor in humans is the manipulation and ligation of the splenic vasculature leading to the development of a low flow “cul de sac” promoting *in situ* thrombus formation (6–8, 10). Intra-abdominal blood stasis has been described in people with splenic masses, especially with larger masses, and may also play a role in post-operative thromboembolism (10). The loss of splenic haematologic filtering following splenectomy may allow cellular debris to persist in circulation, leading to endothelial alterations that may predispose to hypercoagulable states (10). The use of laparoscopy rather than open surgery in people may also contribute to post-operative thrombosis (28, 29). Technical issues related to splenectomy in dogs (e.g., ligation with suture vs. use of electronic vessel sealing devices) may warrant further consideration.

In the current study, significant differences in hemostatic biomarkers were found between dogs with splenic masses and normal dogs. Viscoelastic changes consistent with hypercoagulability were commonly identified in this cohort of hemodynamically stable, non-anemic dogs. Although viscoelastic testing is commonly employed for identification of hypercoagulability in dogs, there are *in vivo* and *ex vivo* limitations to this methodology (30). One such limitation is the concern that lower circulating red blood cell mass leads to artifactual hypercoagulability in dogs due to the dilutional effect of red blood cells on clotting factors (31). To avoid the potential influence of anemia on TEG, non-anemic dogs were enrolled in this study. Recent studies have identified that the fibrinogen concentration, rather than PCV, is the major determinant of the MA on TEG, however (20, 32).

Hypercoagulability may occur secondary to prothrombotic factor excess (e.g., hyperfibrinogenemia), antithrombotic factor deficiency (e.g., antithrombin deficiency), or impaired fibrinolysis. The dogs with splenic masses in this study had significantly decreased AT activity compared to normal dogs. Antithrombin is the most important endogenous anticoagulant, accounting for up to 80% of the inhibitory effect of plasma on coagulation (33). Decreased AT activity may develop secondary to impaired synthesis (e.g., hepatopathy), increased consumption (e.g., active thrombosis), or increased loss (e.g., protein losing nephropathy). Based on the inclusion criteria of the study, physical examination characteristics of the dogs with splenic masses, and results of hemostatic diagnostic tests in the dogs with splenic masses, the most likely explanation for AT deficiency in these dogs was increased consumption. Furthermore, the majority of these dogs that had AT activity below the reference interval also had concurrently elevated plasma TAT concentration (14, 34). Excessive thrombin generation occurs in hypercoagulable states. Any unbound thrombin is rapidly inhibited by AT, resulting in the generation of TAT complexes. Recognition of increased plasma TAT concentrations in individual patients is suggestive of enhanced *in vivo* thrombin generation and clot turnover, supporting the rationale that consumption is the driving process behind AT deficiency in some of these dogs.

Hypercoagulability has previously been reported in dogs and people with neoplasia, particularly those with malignant tumors (12–14). Hypercoagulability in neoplasia is likely multifactorial in nature and may include mechanisms other than endogenous anticoagulant inhibition. Aberrant tissue factor expression has been identified in hemangiosarcoma cell lines previously (35) and there is potential for cross talk between inflammation and hemostasis (36).

Based on the TEG parameter LY60%, normal dogs had significantly greater fibrinolytic activity compared to the dogs with splenic masses in this study. Hypofibrinolysis may play an important role in prothrombotic risk in people (37, 38). Hypofibrinolysis may develop associated with an excess of antifibrinolytic substances, including thrombin-activatable fibrinolysis inhibitor (TAFI) and plasminogen-activator inhibitor 1 (PAI-1). Antifibrinolytic substances have been implicated in thrombosis in people with several diseases, including cirrhotic liver disease and neoplasia (39–42). Likewise, increased concentrations of TAFI have previously been assayed in dogs with malignancy (43). Enhanced *in vivo* thrombin generation in the dogs with splenic masses in this study may have led to increased TAFI production and hence more persistent blood clots. Additional laboratory indicators of fibrinolysis were not specifically evaluated in these dogs. More work on the contribution of hypofibrinolytic states in dogs with thrombosis is needed, especially since no detectable clot lysis on TEG (e.g., LY60% = 0%) may be seen in normal dogs. Adding tissue plasminogen activator to TEG may improve characterization of fibrinolysis in dogs (11, 44). Unfortunately, this was not done in the current study but might be a helpful in future work investigating the role of hypofibrinolysis in dogs.

A limitation of this study was that only a small number of dogs with splenic masses were enrolled, and another is that these dogs were evaluated at a single time point with a limited array of hemostatic tests. It is also a limitation that none of the dogs investigated here ultimately developed detectable or clinically evident post-splenectomy thrombosis. Since portal system thrombosis is an uncommon complication of splenectomy in dogs, a greater number of dogs will be necessary in the future to examine associations between patient characteristics and evidence of thrombosis development. An additional limitation of the study was that specific age, sex, and breed matching in the healthy control population was not performed. Appropriate case matching may have further strengthened the validity of the differences identified between dogs with splenic masses and normal dogs. Lack of routine imaging among the reference population may have resulted in dogs with undetected splenic masses being included in the control population, which has the potential to influence the comparisons drawn here. In this study, ultrasound was employed to identify pre-operative evidence of PST and clear visualization of the portal vein was only possible in 60% of the dogs. Alternative imaging techniques (e.g., computed tomography with contrast) would have a greater sensitivity for identifying thrombosis and would also be more helpful than ultrasound in the recognition of thrombosis in the post-operative period (45). Lack of standardized post-operative imaging may have resulted in underestimation of post-splenectomy thrombosis, particularly microthrombotic events which are often undetectable on clinical examination alone.

The immediate post-operative period may also be quite dynamic and multiple factors could contribute to acute enhancement of thrombogenesis in certain patients. Additional contributors to thrombosis in the post-operative period, specifically the role of vascular stasis and endothelial function, also warrant investigation. Regardless, pre-operative prediction of thrombosis in dogs with splenic masses will likely continue to be difficult. Without an ability to accurately predict thrombosis, the ability to individualize post-operative therapy for dogs (e.g., institution of antithrombotic drugs) will remain challenging.

In summary, pre-operative laboratory abnormalities consistent with the presence of hypercoagulability were common in a population of stable, non-anemic dogs with benign and malignant splenic masses. Endogenous antithrombin deficiency could contribute to the development of hypercoagulability in dogs with splenic masses. Dogs with splenic masses were also significantly hypofibrinolytic compared to a reference population. The contribution of hypofibrinolysis to thrombosis in dogs warrants further investigation. Likewise, additional work focused on dynamic hemostatic and vascular changes occurring in the immediate post-operative period is warranted. Overall, this study provides data to suggest pre-existing systemic hypercoagulability is common in dogs undergoing splenectomy for splenic masses, but its association with post-operative thrombosis development remains undetermined and consideration of post-operative anticoagulant therapy should be based on individual patient risk.

## Data Availability Statement

The raw data supporting the conclusions of this article will be made available by the authors, without undue reservation.

## Ethics Statement

The animal study was reviewed and approved by Clinical Science Research Committee Institutional Review Board, Tufts Cummings School of Veterinary Medicine. Written informed consent was obtained from the owners for the participation of their animals in this study.

## Author Contributions

AL, CW, EM, AdL, and TO'T contributed to conception and study design. AL, TO'T, and JM organized the database and performed statistical analysis. JM wrote the first draft of the manuscript. AL and TO'T wrote sections of the manuscript. All authors contributed to manuscript revision, read, and approved the submitted version.

## Funding

This study was funded by the Companion Animal Health Care Fund and Tufts Cummings School of Veterinary Medicine.

## Conflict of Interest

The authors declare that the research was conducted in the absence of any commercial or financial relationships that could be construed as a potential conflict of interest.

## Publisher's Note

All claims expressed in this article are solely those of the authors and do not necessarily represent those of their affiliated organizations, or those of the publisher, the editors and the reviewers. Any product that may be evaluated in this article, or claim that may be made by its manufacturer, is not guaranteed or endorsed by the publisher.

## Footnotes

^1^Lactate Plus, NOVA Biomedical Corporation, Waltham, MA.

^2^ADVIA 120 Hematology Analyser, Siemens Diagnostics Tarrytown, NY.

^3^TEG 5000 Hemostasis Analyzer, Haemonetics Corp, Braintree, MA.

^4^Kaolin activator, Haemoscope Corporation, Braintree, MA.

^5^ACL-Elite, Beckmann-Coulter, Miami, FL.

^6^Siemens Healthcare Diagnostics Products GmbH, Marburg, Germany.

^7^R package version 3.6.3, Vienna, Austria, 2019.

^8^SAS software v9.4, SAS Institute Inc., Cary, NC.
